# Are recovery stories helpful for women with eating disorders? A pilot study and commentary on future research

**DOI:** 10.1186/s40337-018-0206-2

**Published:** 2018-08-15

**Authors:** Lisa Dawson, Barbara Mullan, Stephen Touyz, Paul Rhodes

**Affiliations:** 1 Eating Disorder Service, The Sydney Children’s Hospital Network, Westmead Campus, Sydney, Australia; 2 Centre for Family Based Mental Health Care, St Vincent’s Private Hospital, Sydney, Australia; 3 School of Psychology and Speech Pathology, Curtin University, Perth, WA Australia; 4 School of Psychology, The University of Sydney, Sydney, Australia

**Keywords:** Eating disorders, Anorexia nervosa, Recovery, Recovery stories, Lived experience

## Abstract

**Background:**

Anecdotally it is well known that eating disorder memoirs are popular with people with anorexia nervosa and recovery stories are readily available online. However, no research to date has empirically explored whether such stories are helpful for current sufferers. The aim of the current pilot study was to explore the efficacy of recovery narratives as a means of improving motivation and self-efficacy and to qualitatively explore patient perspectives of such stories.

**Method:**

Fifty-seven women with anorexia nervosa and subclinical anorexia nervosa participated in this online study. Participants were randomised to either receive recovery stories or to a wait-list control group. After completing baseline measures, participants read five stories about recovery, and completed post-intervention measures two weeks later.

**Results:**

The quantitative results indicated that reading stories of recovery had no effect on motivation and self-efficacy over a two-week period. In contrast, the qualitative results showed that the stories generated thoughts about the possibility of recovery and the majority indicated they would recommend them to others.

**Conclusions:**

This study adds to a growing body of research exploring the integration of voices of lived experience into treatment approaches. Future research should focus on 1) identifying for whom and at which stage of illness recovery stories might be helpful; 2) the mechanism via which they might operate; and 3) the most helpful way of presenting such stories.

## Background

Traditional treatments for those with anorexia nervosa have not been wholly effective [1] and, accordingly, new treatment approaches need to be explored. The Recovery Model, which emerged from consumer movements (emphasising the personal experience of recovery, involving hope, connection, and establishing a personally fulfilling life) [2], has emerged as an alternative means to consider treatment approaches for people with eating disorders [3]. This model privileges the sharing of consumer knowledge and experience as an important part of recovery and can include ways for peers to be involved in treatment [4]. While there are few consistent definitions of recovery in the field of eating disorders [5], historically, recovery has been defined using medical model approaches including varying composites of physical, behavioural, and, more recently, psychological domains [5, 6]. Such definitions neglect domains that may encompass more general well-being or quality of life hence the move to embrace more holistic and person-centred approaches [3]. While recovery-oriented care has been identified by the Australian Health Ministers’ Advisory Council and the NSW Mental Health Commission as a priority in order to create modern, open and inclusive mental healthcare services [7] few treatment approaches have yet been developed to provide this and there is little research exploring approaches to treatment that include the sharing of lived experience. However, these ‘interventions’ are happening in the consumer community anyway. One such example of this is the sharing of personal stories of recovery.

In the last two decades there has been a significant increase in the number of published eating disorder memoirs [8] and recovery stories are readily accessible on the internet via eating disorder organisation websites, blogs, online forums, social networking sites, and video channels with the authors detailing the experience of living with and recovering from an eating disorder. Anecdotally it is well-known that people with eating disorders often read recovery stories; however, there is a significant absence of research in this area and it remains unknown how helpful they are considered by current sufferers. While narrative therapists have used stories to encourage alternative voices to the voice of anorexia nervosa [9] and have attempted to circulate liberating stories of recovery via the ‘Archives of Resistance’, a website dedicated to sharing ‘anti-anorexia’ stories [10] the efficacy of such an approach has not been tested. A single laboratory study has been conducted on recovery stories which explored the effect of reading eating disorder memoirs on 50 undergraduate students with no eating disorder pathology [8]. This study found that eating disorder memoirs had no effect on the eating attitudes and behaviours of participants, however, the experience of reading recovery stories for people with an eating disorder remains unknown and questions remain as to their potential effects.

The aim of the current study was to explore the effect/s of reading recovery stories for women with a current eating disorder, specifically exploring how helpful they are. There are inherent complexities when opening up a new area of research and given the dearth of research in this area knowing what (and how) to explore is complex. As such, we first look to literature in the field more broadly to determine any preliminary hypotheses as to why and how recovery stories might be helpful or unhelpful for readers. Firstly, recovery stories might offer a means of increasing motivation and self-efficacy. Ambivalence about recovery is consistently identified as one of the major challenges to treatment [11, 12] and a lack of self-efficacy is associated with poorer outcome [13]. Patients with anorexia nervosa have been found to perceive the illness as having low controllability and curability, and as chronic [14]. Given that perceiving recovery as impossible has been identified as a core obstacle to change [15, 16] people with anorexia nervosa might benefit from knowing that people can and do recover [17].

A second mechanism through which such recovery stories could be helpful is the provision of information about different pathways to recovery. Patient-centred accounts that are strengths-focused (as opposed to deficits-focused) and offer examples of how individuals move from illness to wellness can impart guidance [18], potentially increasing awareness and understanding about the process of recovery [18]. Additionally, those with lived experience are in a unique position to offer hope, understanding, solidarity, and vital information. This allows for the sharing of ‘insider knowledge’ [19] which can be more influential and empowering than messages delivered by health professionals [20]. Delivering messages in narrative format may also mean that messages are less likely to be rejected by readers than other forms of motivational messages [21]. It has been proposed that narrative forms of communication reduce counter-arguing and facilitate observational learning [21]. Narratives have been used to enhance patient well-being and healthcare participation for cancer patients [22] and narrative information has positive effects on health care participation [23]. It has been suggested that patient narratives might be more effective forms of communication than didactic formats (e.g., fact sheets, brochures) because they are more engaging and provide role models [23].

Despite these conceivable benefits, there is also the possibility that stories of recovery might be unhelpful due to potential iatrogenic effects [8] as anorexia nervosa may be associated with social learning processes of imitation, identification and competition; a so-called ‘peer contagion’ effect [24]. Patients have reported that group treatment settings can be a competitive environment [25] leading to new eating disordered habits [26]. Similarly, popular pro-eating disorder online communities [27], are believed to be potentially harmful due to the exchanging of inappropriate health messages and that people access such sites to learn ‘how to be anorexic’ [28]. However, one of the main attractions to visiting pro-eating disorder websites is in fact the seeking of support and belonging [27]. These sites offer support that is not available from regular face-to-face contact [29]. Recovery stories delivered responsibly might be able to offer a similar supportive function while limiting the potential dangers.

The aim of this pilot study was to explore the effect of recovery stories on a clinical sample by firstly exploring if they have any effect on improving motivation and self-efficacy. Recovery stories were compared to a wait-list control group and the primary outcome variable was change in motivation as measured by (i) intentions to recover and (ii) stage of change. Secondly, this study aimed to qualitatively explore the experience of reading such stories for women with eating disorders.

## Method

### Quantitative design

A randomised, waitlist control trial design was used. After completing the screening measure to assess for eligibility, participants were randomly allocated to either the intervention (reading recovery stories) or waitlist control group. Randomisations were conducted using an online random number generator. Consistent with a truly random sequence of numbers, it was not specified that there should be an equivalent number of participants in each condition. Participants were randomised according to the order in which they were eligible to participate, in order to limit selection bias. Allocation was single blinded (concealed from participants, but not researchers). The intervention was conducted according to the protocol approved by the University Human Research Ethics Committee and was registered as ACTRN12613000795796.

### Qualitative design

Thematic analysis was used in the current study. This method allows for identifying, analysing and reporting patterns (themes) within data [30]. Given the lack of existing theory or research on the phenomenon under investigation, an inductive approach was used in that themes were strongly linked to the data (rather than pre-existing theory) [31]. This form of thematic analysis is data-driven [30] and one benefit to such an approach is that preconceived categories or theoretical perspectives are not imposed upon the data.

### Participants

Participants were recruited over a nine-month period by advertising details of the study on a number of eating disorder websites in Australia (e.g., National Eating Disorder Collaboration) and the UK (e.g., B-EAT UK), blogs (e.g., junealexander.com, dropitandeat.blogspot.com.au) and other social media platforms. Individuals with all forms of clinically significant anorexia nervosa-type eating disorders were recruited. Inclusion was determined based on the responses to self-report questions pertaining to the anorexia nervosa diagnosis from the Mini International Neuro-Psychiatric Interview (MINI [32];) as well as current self-reported BMI. Self-reported height and weight has been found to derive accurate BMI data [33]. Participants were required to meet the following inclusion criteria: be aged at least 18 years, and fulfil research criteria for anorexia nervosa phenotype, which were: (i) meeting criteria A and B of DSM-IV for AN (i.e. refusal to maintain a normal body weight and intense fear of weight gain); and (ii) BMI < 20 kg/m^2^. Those with subclinical anorexia nervosa were included in order to assess if the intervention was effective across all clinically significant forms of anorexia nervosa.

### Intervention (stories of recovery)

The recovery stories included the narratives of five Australian women who had fully recovered from severe and enduring anorexia nervosa. The narratives were developed following in-depth interviews with the recovered women about the process of developing, living with, and eventually recovering from severe and enduring anorexia nervosa, as part of a previous study [16]. The stories focussed mainly on the recovery process. The narratives were written in consultation with the recovered women and averaged 5000 words. Details in the stories that were regarded as specifically triggering, such as reporting weights, body mass indexes, or specific means for weight loss, were removed from the stories. In order not to deviate too much from the women’s representation of their experience, and to replicate the kinds of stories that might be accessible to eating disorder sufferers, the stories were not edited further.

The stories were chosen to be maximally divergent, in order to represent the breadth of recovery experiences. The women developed anorexia nervosa between the ages of 11 and 15. Three were recovered by the time they were in their mid-to-late twenties, one recovered in her thirties, and one in her fifties. One of the women had experienced childhood sexual abuse. Some found treatment helpful, others recovered without professional support. A variety of treatment interventions were reported including hospitalisation, inpatient treatment, outpatient treatment, medical treatment, psychotherapy, and alternative treatments. The narratives were not designed to be prototypical recovery stories; rather they reflected the real experiences of these women. In addition to the recovery stories, participants were provided with information about eating disorder support services. Participants were informed that the stories were not intended to be a substitute for professional intervention and participants were encouraged to seek professional support where needed.

#### **Measures.** Screening and demographics

At baseline, participants completed measures of demographics and eating disorder history. The following questionnaires were administered at baseline and post-intervention.

### Motivation

The Anorexia Nervosa Stages of Change Questionnaire [34] was used to measure motivation via stage of change. It is a 20-item measure that assesses readiness to recover as measured by stage of change according to the Transtheoretical Model of Change [35]. Each item refers to a specific anorexia nervosa symptom and contains five statements representing the stages of change (e.g. pre-contemplation, contemplation,). For each item the individual is asked to select the statement that best describes their current attitude or behaviour (1–5). The total readiness to change score represents the sum of all 20 items (range: 5–100), which is divided by the number of items to obtain the stage classification score. The measure has demonstrated internal consistency, test-retest reliability and convergent, discriminant, and concurrent validity [34, 36].

The Predicting Intentions to Recover from anorexia nervosa questionnaire [37] was used to measure intention to recover. Based on the theory of planned behaviour [38], this 25-item questionnaire measures attitudes, subjective norm, perceived behavioural control, and intention, associated with recovery on a 100-point sliding scale. Subscale scores represented the weighted sum of all relevant items and higher scores in each case indicated more positive constructs. Unlike the ANSOC-Q, this measure includes items assessing self-efficacy (perceived behavioural control scale).

An additional measure of motivation used was the Treatment Self-Regulation Questionnaire (TSRQ [39];) (adapted for anorexia nervosa). Based on Self-Determination Theory [40], this measure assesses how and why people engage in a healthy behaviour or try to change an unhealthy behaviour. The TSRQ contains 15 items and assesses different forms of motivation: autonomous, controlled and amotivation. A seven-point Likert scale ranging from 1 (not at all true) to 7 (very true) is used. Each motivational type composes a subscale of the measure. It has been successfully adapted to a number of different health behaviours and is validated [41]. The measure has reasonable validity and reliability to assess motivation and the internal consistency of each subscale has been found to be acceptable [41].

Finally, six self-report likert scales were used to assess motivation and self-efficacy based on questions used by Wade and colleagues [42]. These questions assessed how important, confident and ready participants felt about recovering from their eating disorder and eating normally and gaining weight. Participants rated their response on a 10-point Likert scale (from 1 = not at all to 10 = very) with higher scores indicating greater levels of importance, confidence and readiness [42].

### Eating pathology

The Eating Disorder Examination-Questionnaire (EDE-Q [43];) is a self-report questionnaire measure of eating disorder psychopathology, which was adapted from the Eating Disorders Examination [44]. It contains 36-items across four subscales. Each item is rated on a seven-point scale (0–6). Subscale scores range from 0 to 6; a total score can also be computed by summing the subscale scores and dividing by four (range = 0–6). The EDE-Q has been validated in community samples and has demonstrated robust psychometric properties. The EDE-Q was adapted to measure a two-week period for the purposes of this study.

### Psychological symptoms

The Depression, Anxiety, and Stress Scale (DASS 21 [45];), is a 21-item self-report measure. Each item is rated on a scale from 0 (did not apply to me at all) to 3 (applied to me very much or most of the time); Subscale scores range from 0 to 42, with higher scores indicating more severe/frequent symptoms. The DASS has strong psychometric properties [46]. The Positive and Negative Affect Scale (PANAS [47];) is a 20-item self-report questionnaire that assesses current state mood. It includes 10 items relating to positive affect (e.g., enthusiastic, excited) and 10 items relating to negative affect (e.g., hostile, distressed). Each item is rated on a five-point scale from 1 (slightly or not at all) to 5 (extremely). Higher scores indicate higher levels of positive and negative affect respectively. The PANAS is a valid and reliable measure of affective responses [48].

### Qualitative feedback and adherence

Post-intervention, participants were asked adherence questions to confirm whether the intervention materials had been read. Qualitative feedback was also collected in which participants were asked nine open-ended questions about the experience of reading the stories. Questions included were What was the experience of reading the stories like for you? What interested you? What kinds of things did it get you thinking about? What was helpful? What was unhelpful? How relevant were the stories for you? Would you recommend recovery stories for others with eating disorders? Why or why not? How could the stories be made more helpful for those with eating disorders?

### Procedure

The study was completed online via Qualtrics Survey software. Participants who inquired about the study received information about access to support services for eating disorders at initial contact. Participants were emailed a link to the eligibility questionnaire. Eligible participants were individually randomised and were blind to their allocation. Once baseline measures were completed participants in the intervention group were emailed the *Stories of Recovery* booklet and were asked to read it over the next two weeks. Two weeks after the completion of baseline measures, participants completed outcome measures. Those in the waitlist control group repeated the baseline measures two weeks after their initial completion and were then sent the *Stories of Recovery*. Two weeks later they were sent the final outcome measures.

### Data analysis

#### Intervention outcome

A mixed between-within participants ANOVA was conducted to assess the impact of the intervention (stories of recovery) on participants motivation between groups (experimental and control) and across two time periods (pre and post intervention). The primary outcome of interest was change in motivation as measured by the ANSOC-Q, the TPB measure, the TSRQ, and the SRL. Secondary outcomes were change in eating disorder psychopathology (EDE-Q), positive or negative affect (PANAS), and psychological symptoms (DASS). Tests of the 15 dependent variables were conducted using a Bonferroni adjusted alpha level of .003 (.05/15) to control for family-wise error.

#### Qualitative data

Thematic Analysis was used to derive themes that characterised the experience of reading the stories of recovery. Based on guidelines for analysing qualitative data using Thematic Analysis [30], all open-ended responses were read carefully from beginning to end to achieve immersion and attain a sense of the data. Responses were then re-read to derive codes. Each question was analysed independently. Exact words from the text that appeared to encapsulate key concepts were highlighted and used to describe themes. Throughout this process common themes emerged and were grouped using an inductive approach. To ensure rigour an audit trail was maintained throughout [49] and all data was cross-coded by two of the authors (LD and PR) with experience in qualitative research. Using participants own words in the coding process added to the trustworthiness of the data and led to a high level of agreement between coders in regard to themes.

## Results

### Participants and response rate

Fifty-seven women completed measures at baseline. Of these twenty-six were randomised to the experimental group (45.6%) and 31 randomised to the control group (54.4%). Eight participants (14%) did not complete the study. Participants ranged in age from 19 to 70 years (average age = 29, SD = 10.2). The average BMI was 16.85 (range 11.1–20; SD = 1.92). On average, participants had had an eating disorder for 13 years (range 1–55 years; SD = 9.88). Ninety-five percent of the sample would have met diagnosis for anorexia nervosa according to DSM-5 criteria. Twenty-three participants were from Australia (40.4%), 16 from the UK (28.1%), 16 from the USA (28.1%), and 2 from Canada (3.5%). Overall the sample was well educated, with 82.5% having completed education beyond high school. The majority of the sample (75.4%) was receiving some form of treatment for an eating disorder at the time of the study.

### Baseline differences between groups

The results indicated that the two conditions did not differ significantly at baseline in terms of age or length of illness (*p* > .05). There was a difference between the groups in terms of BMI (*p* < .05) where individuals in the experimental group had lower average BMI (*M* = 16.23, *SD* = 1.9) compared to the control group (*M* = 17.38, *SD* = 1.8). There were no differences between groups on other variables (*p* > .05).

### Descriptive statistics

Table 1 shows the mean scores for all of the variables. For positive affect (*M* = 22.5) participants were within 1 *SD* of norms (*M* = 29.7, *SD* = 7.9). For negative affect (mean = 26.0) participants were more than 1 *SD* away from the mean (*M* = 14.8, *SD* = 5.4) [47]. The mean scores for each of the DASS subscales (depression, anxiety, and stress) fell into the extremely severe range [45]. The mean score for eating disorder psychopathology (*M* = 4.17, *SD* = 1.23) placed participants well above community norms (*M* = 1.554, *SD* = 1.213) [50]. The mean score for the ANSOC-Q placed participants in the preparation stage of change [34].

**Table 1:** Mean scores for positive and negative affect, depression, anxiety, stress, eating disorder psychopathology, stage of change, TPB, TSRQ, SRL for the baseline sample (*N* = 57)

Variable	Mean	*SD*	Range
Positive affect (PANAS)	22.5	6.7	11–40
Negative affect (PANAS)	26.0	9.78	12–48
Depression (DASS)	18.32	6.04	7–28
Anxiety (DASS)	15.44	5.01	7–26
Stress (DASS)	19.04	4.57	9–28
ED psychopathology EDE-Q	4.17	1.23	.82–6
Stage of change (ANSOC-Q)	2.64	.77	1.25–4.35
Intention (TPB)	60.64	18.1	12.5–98.25
Attitude (TPB)	70.3	15.7	31–98.86
Subjective Norm (TPB)	56.3	27.0	0–88.33
PBC (TPB)	42.05	16.67	4.36–70.91
Autonomous motivation (TSRQ)	4.75	1.42	1.67–7
Controlled motivation (TSRQ)	4.51	1.39	1.67–6.83
Amotivation (TSRQ)	2.78	1.09	1–5.67
Self-reported motivation	5.7	2.19	1.17–9.67

Note: PANAS higher scores indicate higher levels of positive and negative affect, range 10–50, positive affect *M* = 29.7, *SD* = 7.9, negative affect *M* = 14.8, *SD* = 5.4; DASS higher scores indicate more distress, range for each subscale = 0–21; EDE-Q higher scores indicate higher levels of eating disorder psychopathology, range = 1–6; ANSOC-Q higher scores indicate higher levels of motivation, range = 1–5; TPB range for each composite = 1–100, higher scores indicate more positive intentions/beliefs and greater perceptions of normative pressure and control; TSRQ range for each subscale = 1–7, higher scores indicate higher levels of motivation type; self-reported motivation range = 1–10, higher scores indicate higher levels of motivation

### Test of representativeness

#### Drop-out attrition

The results indicated that participants who completed the intervention were not significantly different to those who dropped out on any measures (all *p* > .05).

### Intervention outcome

#### Primary outcome: Motivation

There were no significant differences between groups over time on any of the primary outcome variables (see Table 2). Specifically, there was no significant interaction between group and time for stage of change [ANSOC-Q; *M = F* (1, 47) = 2.83, *p* > .003; attitude *F* (1, 47) = .0.45, *p* > .003; subjective norm *F* (1, 47) = 2.54, *p* > .003; perceived behavioural control *F* (1, 47) = .083, *p* > .003; or intention *F* (1, 47) = .66, *p* > .003. There was no significant difference between group and time for TSRQ autonomous motivation *F* (1, 47) = .179, *p* > .003; controlled motivation *F* (1, 47) = .087, *p* > .003; or amotivation *F* (1, 47) = 1.51, *p* > .003 or for self-reported motivation *F* (1, 47) = .3.085, *p* > .003.

**Table 2:** Mean scores for main variables at baseline and follow up (*N* = 57)

Variable	Baseline	Follow-up
Stage of change (ANSOC-Q)	2.64 *(.77)*	2.76 *(1.0)*
Intention to recover (TPB total score)	56.03 *(14.5)*	57.84 *(17.1)*
Autonomous motivation (TSRQ)	4.75 *(1.42)*	4.7 *(1.6)*
Controlled motivation (TSRQ)	4.51*(1.39)*	4.26 *(1.65)*
Amotivation (TSRQ)	2.78 *(1.09)*	2.69 *(1.3)*
Self-reported motivation	5.7 *(2.19)*	5.58 *(2.6)*

#### Secondary outcomes

There were no significant differences between group and time for positive *F* (1, 47) = .158, *p* > .003 and negative affect *F* (1. 47) = .899, *p* > .003 or eating disorder psychopathology *F* (1, 47) = 1.7, *p* > .003. On the DASS subscales there were no interaction effects for depression *F* (1, 47) =3.23, *p* < .003 or anxiety *F* (1, 47) = .78, *p* < .003; or stress *F* (1, 47) = 4.04, *p* > .003.

### Qualitative results

#### Overview of findings

Thirty-eight participants responded to nine open-ended questions. Common themes identified across questions and participants were that the stories generated feelings of hope, inspiration, and of being understood. The ‘possibility of recovery’ was a recurring theme across questions and participants. The two most widely endorsed themes across all the qualitative data were in response to the questions “What kinds of things did the stories get you thinking about?” and “What was helpful about the stories?” The majority of the sample reported that the sense that recovery was possible was most helpful, however, approximately one quarter of participants also reported that the stories could be triggering and result in the reader making unhelpful comparisons between themselves and the individuals in the narratives. Such comparisons included thinking “I’m not sick enough”, or “not being good enough” at anorexia nervosa.

#### What was the experience of reading the stories like for you?

A variety of themes were identified with some participants reporting that the experience was both simultaneously positive and negative: “sometimes upsetting; sometimes inspiring”. Positive experiences included hope, inspiration, and feeling understood and less isolated. Negative responses included finding the stories triggering, unhelpfully comparing oneself to the individuals in the stories, and being disheartened or saddened by the aspects of the stories when the author was in the grips of the eating disorder.

#### What interested you?

Participants were interested in the inspirational nature of the stories, the possibility of recovery after such a long duration with anorexia nervosa, and strategies used for recovery: “What interested me were the different specific strategies that supported the women to recover, e.g. finding the right team and trusting that team instead of the eating disorder”. Some participants reported that the variation across stories demonstrated that there was not a “one size fits all approach to recovery” others noted the similarities between themselves and the authors.

#### What kinds of things did it get you thinking about?

There was less variation in responses to this question with the majority of participants reporting that the stories generated thoughts about the possibility of recovery, what it takes to recover, things they would like to change, and life after an eating disorder (Table 3).

**Table 3:** Example responses to “What kinds of things did it get you thinking about?”

It made me think about where I am in my recovery and the aspects I need to challenge.	
How I could move forward even though I feel hopeless at the moment.	
It got me thinking about the devastating effects of this disease and how enduring or long lasting it can be. It got me thinking that I didn’t want to be 50 years old, or even 30 years old with children and still be battling an eating disorder every day.	
That recovery is possible.	
Recovery is possible and the ways in which each manages it.	
The things that do and don’t help people (and how widely that can vary). The importance of positivity and determination	
Why I am choosing recovery what life is like without an eating disorder, that I want to live rather than exist, that thoughts are only thoughts and that fat is not a feeling. It reminded me of the tools I need to use to recover and not to be so self-critical when I have a bad day or make a mistake. It reminded me of my health and the damages that come from having an eating disorder. It made me want to start planning and thinking about my treatment and action plan to get back on track.	
That I need to stop saying I’ll do it tomorrow and not let it go any further because a full recovery is possible and worth it.	
Why I want to get better and my recovery journey in general.	
What it takes to recover.	
That everyone has hard times but with the right help and support, anyone can overcome and beat this disease.	

#### How relevant were the stories for you?

Most participants found relevance in the stories with 13 stating that the stories were very relevant and 18 stating somewhat relevant. Seven participants considered them not relevant.

#### Would you recommend recovery stories for others with eating disorders? Why or why not?

The majority of participants reported they would recommend recovery stories for others with eating disorders: “Yes. Reading stories of recovery can provide hope that you are not alone and that you have the ability to recover as well”. Some participants reported that their recommendation would be dependent on the individual or on the stage of recovery of an individual. Only three participants reported that they would not recommend recovery stories.

#### What was helpful and/or unhelpful?

Again, there was a strong endorsement by more than half of the participants that the sense that recovery was possible was helpful. Some participants reported that the stories made them feel understood and less alone. In terms of what was unhelpful, the most strongly endorsed responses were that the stories could be triggering.

#### How could the stories be made more helpful for those with eating disorders?

Some participants reported that having more stories or an increase in story variation would be helpful, including stories of individuals with a later onset eating disorder, shorter duration of illness, and the stories of men.

## Discussion

The current study provided the first exploration of the effect of recovery stories on a clinical sample. The quantitative results indicated that reading stories of recovery had no effect on improving motivation and self-efficacy over a two-week period. Additionally, there were no significant differences between groups post intervention on any of the secondary outcome measures. In contrast the qualitative results showed that the stories generated thoughts about the possibility of recovery and the majority of participants would recommend recovery stories to others.

Although the current study found recovery stories exhibited no quantitative effect on motivation or self-efficacy, these null results should be interpreted with caution. There are some important design features of the current pilot study that might explain these findings. First, the intervention dose was small. Five stories were chosen as it was thought this was an achievable number to ask individuals to read over a two-week period, however a more intense intervention may be needed. The stories might have had more effect if they included guided activities/reflections or were integrated into a therapy program. Equally, it is possible that the specific stories used in the current study were ineffective. Further, it is also possible that participants experienced changes in constructs not directly measured in this study, or that changes surfaced after the two-week follow-up. Additionally, the characteristics of the sample need to be considered when interpreting the results. For example, how participants were recruited might have resulted in a self-selecting sample. These individuals might already have been familiar with recovery stories, which could have resulted in a saturation effect, and this should be controlled for in future research. Participants also scored high on measures assessing eating disorder and psychological symptoms and might have been too unwell to benefit from the stories.

Another reason why the quantitative results should be cautiously interpreted is the discrepancies between the quantitative and qualitative data, with qualitative data mainly supporting benefits but quantitative data not showing any improvements. Potentially future research could explore variables in addition to motivation and self-efficacy. Participants reported that the stories resulted in increasing feelings of hope and reducing feelings of isolation; exploring the effect these variables in future research would therefore be useful. The qualitative results might also be indicative of broader changes that the current design did not capture and are not easily measureable with current research methodologies. For example, the reading of recovery stories might be akin to planting a small seed that leads to changes that emerge slowly over time, thus follow-up measures might be useful in future studies.

In addition to finding the stories helpful in terms on increasing solidarity a minority of participants also reported that they could be unhelpful. Despite efforts to remove any obviously triggering material from the stories, some participants reported being triggered by the narratives. While there is individual variation in what will be considered triggering, a challenge for developing recovery stories will be balancing the need to present stories responsibly with the need to present an authentic account that does not necessarily skirt around the challenges of illness and recovery. Stories need to be relatable to be effective [51], so it is likely that recovery stories need to include some description of thoughts and feelings associated with an eating disorder so that readers can identify with the struggle. The stories used in the current study reflect the true accounts of five women’s recovery, however, stories that are developed with the specific intention of being a therapeutic tool, for example by having less detail of illness and even more of a recovery focus, might be more helpful in the future.

The qualitative data suggested that recovery stories might help to increase hope and solidarity, which is line with approaches which aim to integrate the voices of lived experience into treatment, thus the findings of this study add to a growing body of research supporting recovery-oriented approaches to mental health. Historically, treatment approaches in the eating disorders have focused more on symptom reduction with less importance placed on solidarity, feeling understood, hopefulness, and the reduction of isolation [3], however, such factors have been continually highlighted as important by those with lived experience of eating disorders [16]. Over the last few decades there has been a move to recovery-oriented care with reforms in mental health reflecting this. The growing recognition and interest in integrating lived experience into treatment has resulted in the uptake of peer roles in consumer advocacy, consumer operated services, and traditional statutory mental health services [52, 53] It has been argued that employing peers with lived experience improves mental health care and service provision [53]. Peer work has been identified as having multiple benefits including the opportunity to connect with a person with lived experience of mental illness and stigma, the sharing of a common language, and facilitating recovery [52]. Additionally, peer work has been associated with the normalising and acceptance of emotional responses associated with mental ill-health [52]. Challenges remain however around training, supervision, and management and many questions about peer work remains [52].

While approaches to treatment which include lived experience have grown in recent years in mental health approaches more broadly, for example the introduction of Peer Supported work in Open Dialogue [54], such approaches have yet to be fully developed in the field of eating disorders. Some approaches currently available in the field include Multi-Family Therapy for young people with an eating disorder and their family [55, 56]; a treatment in which the sharing of support and solidarity amongst families is explicitly prioritised and parent-to-parent consultations in Family-Based Treatment where parents who have ‘graduated’ from therapy consult with parents earlier in the process. Peer-mentoring is also the subject of emerging research in the field, where a person who has experienced recovery mentors a person with a current eating disorder. Early research in this area suggests, in line with the findings from the current study, that peer-mentoring can increase hope for current sufferers [57, 58]. A recent study has examined the effect of therapists working in the field of eating disorders with lived experience of an eating disorder themselves and found that using experiential knowledge can have advantages such as providing insights into the recovery process and enhancing hope for recovery but that this knowledge must be shared thoughtfully and without specific details about symptomatology [59]. The current study adds to the emerging research in the field of eating disorders regarding recovery-oriented approaches and lived-experience integration however, much more research is needed. Determining ways to effectively integrate these approaches into current treatments and broadening treatment targets to include this is needed. The results of this study suggest that the voices of those who have recovered can provide support and understanding and reduce isolation, however, a small minority also reported finding the experience triggering therefore, further research is needed is consider how to best develop and integrate these voices into treatment.

While the current pilot study has contributed to furthering knowledge about the use of recovery stories, there are some limitations. Participants were recruited over the internet and clinical information was self-reported. The two-week time period used in the current study is a very short time frame in which to assess change in symptoms or clinical features. The recovery stories may have had a longer-term effect medicated by an increase in hope. An additional limitation is the lack of an apriori power analysis. Given this is a novel study, clinical effect size could not be determined and, therefore, a power analysis could not be completed. Lastly, given the design, a blinded outcome was not possible. We went to the literature to consider why and how recovery stories might be helpful, however, asking consumers is an important first step, and qualitative research methods might best achieve this aim. Narrative methods might be an appropriate means to explore how recovery stories might effect change and their relationship to the reader. Later research could focus on 1) identifying for whom and at which stage of illness recovery stories might be helpful; 2) the mechanism via which they might operate; and 3) the most helpful way of presenting such stories. There are also likely to be age, cultural, and gender differences in outcomes, which the current study did not explore, which needs to be a focus of future research.

## Conclusion

The findings from the current study suggest that the experience of reading recovery stories is complex. Much remains unknown about how such stories might function for the reader. Given the likelihood that those with eating disorders will be exposed to such stories it is important to more clearly understand the impact of recovery stories. As research and treatment approaches expand to include and prioritise increasing hope, reducing isolation and increasing feelings of solidarity, this study adds to the body of growing research into how to integrate the voices of lived experiences into treatment. This study raises many questions that are needed for future research and may provide an important starting off point as lessons learnt for researching this complex and novel area.
